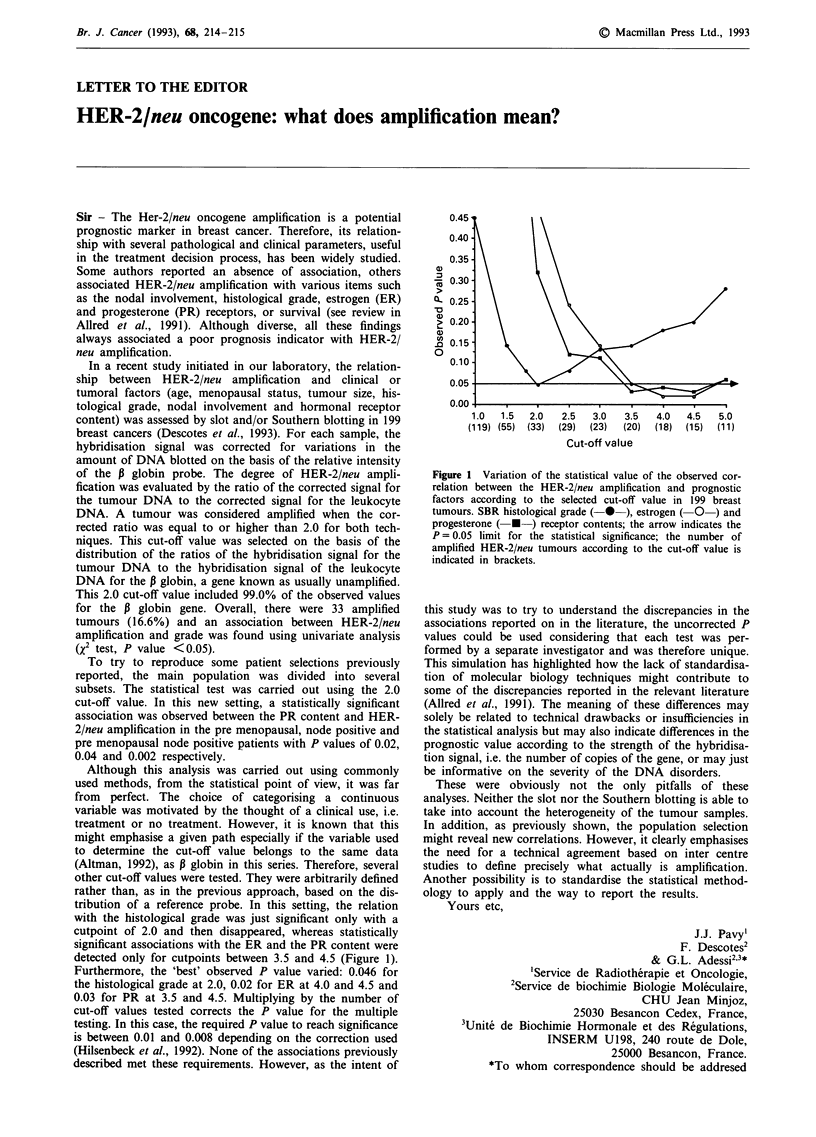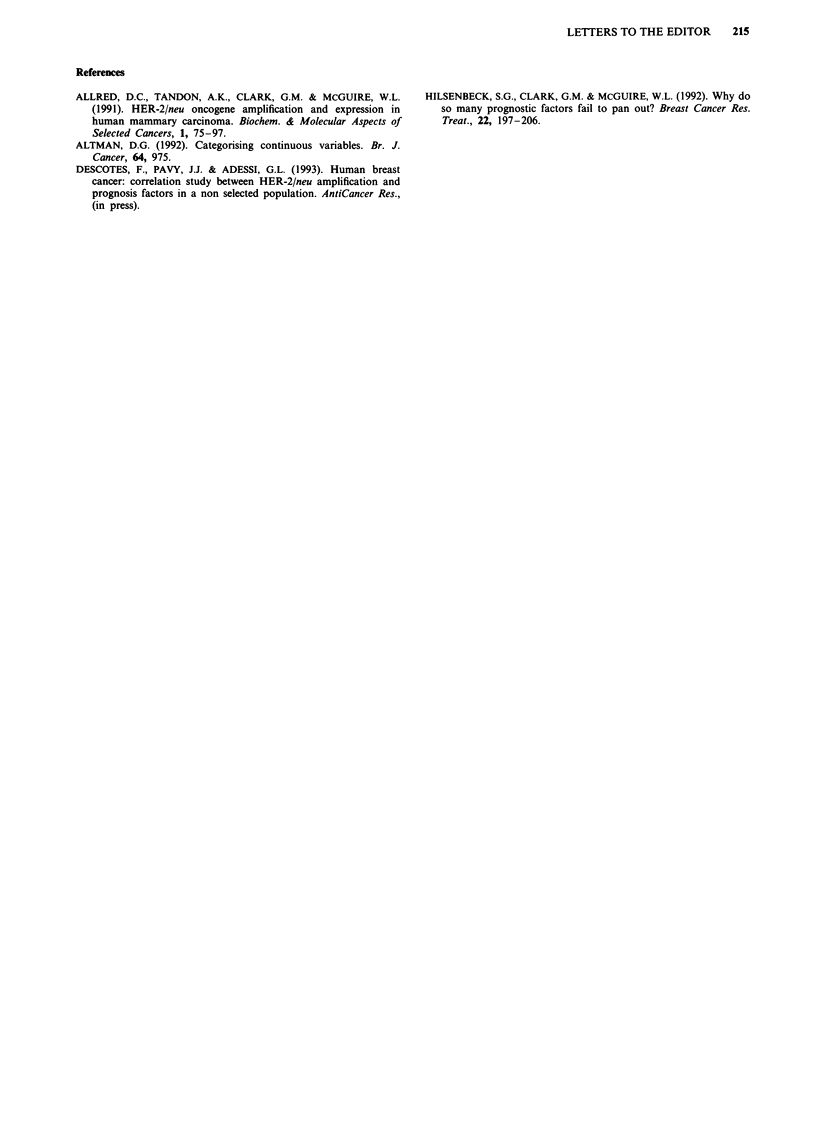# HER-2/neu oncogene: what does amplification mean?

**DOI:** 10.1038/bjc.1993.317

**Published:** 1993-07

**Authors:** J. J. Pavy, F. Descotes, G. L. Adessi


*Br. J. Cancer* (1993), **68**, 214–215 © Macmillan Press Ltd., 1993

### LETTER TO THE EDITOR

### HER-2/*neu* oncogene: what does amplification mean?

**Sir** – The Her-2/*neu* oncogene amplification is a potential prognostic marker in breast cancer. Therefore, its relationship with several pathological and clinical parameters, useful in the treatment decision process, has been widely studied. Some authors reported an absence of association, others associated HER-2/*neu* amplification with various items such as the nodal involvement, histological grade, estrogen (ER) and progesterone (PR) receptors, or survival (see review in Allred *et al.*, 1991). Although diverse, all these findings always associated a poor prognosis indicator with HER-2/*neu* amplification.

In a recent study initiated in our laboratory, the relationship between HER-2/*neu* amplification and clinical or tumoral factors (age, menopausal status, tumour size, histological grade, nodal involvement and hormonal receptor content) was assessed by slot and/or Southern blotting in 199 breast cancers (Descotes *et al.*, 1993). For each sample, the hybridisation signal was corrected for variations in the amount of DNA blotted on the basis of the relative intensity of the $\beta$ globin probe. The degree of HER-2/*neu* amplification was evaluated by the ratio of the corrected signal for the tumour DNA to the corrected signal for the leukocyte DNA. A tumour was considered amplified when the corrected ratio was equal to or higher than 2.0 for both techniques. This cut-off value was selected on the basis of the distribution of the ratios of the hybridisation signal for the tumour DNA to the hybridisation signal of the leukocyte DNA for the $\beta$ globin, a gene known as usually unamplified. This 2.0 cut-off value included 99.0% of the observed values for the $\beta$ globin gene. Overall, there were 33 amplified tumours (16.6%) and an association between HER-2/*neu* amplification and grade was found using univariate analysis ($\chi^2$ test, $P$ value $<0.05$).

To try to reproduce some patient selections previously reported, the main population was divided into several subsets. The statistical test was carried out using the 2.0 cut-off value. In this new setting, a statistically significant association was observed between the PR content and HER-2/*neu* amplification in the pre menopausal, node positive and pre menopausal node positive patients with $P$ values of 0.02, 0.04 and 0.002 respectively.

Although this analysis was carried out using commonly used methods, from the statistical point of view, it was far from perfect. The choice of categorising a continuous variable was motivated by the thought of a clinical use, i.e. treatment or no treatment. However, it is known that this might emphasise a given path especially if the variable used to determine the cut-off value belongs to the same data (Altman, 1992), as $\beta$ globin in this series. Therefore, several other cut-off values were tested. They were arbitrarily defined rather than, as in the previous approach, based on the distribution of a reference probe. In this setting, the relation with the histological grade was just significant only with a cutpoint of 2.0 and then disappeared, whereas statistically significant associations with the ER and the PR content were detected only for cutpoints between 3.5 and 4.5 (Figure 1). Furthermore, the 'best' observed $P$ value varied: 0.046 for the histological grade at 2.0, 0.02 for ER at 4.0 and 4.5 and 0.03 for PR at 3.5 and 4.5. Multiplying by the number of cut-off values tested corrects the $P$ value for the multiple testing. In this case, the required $P$ value to reach significance is between 0.01 and 0.008 depending on the correction used (Hilsenbeck *et al.*, 1992). None of the associations previously described met these requirements. However, as the intent of

**Figure 1** Variation of the statistical value of the observed correlation between the HER-2/*neu* amplification and prognostic factors according to the selected cut-off value in 199 breast tumours. SBR histological grade (—●—), estrogen (—O—) and progesterone (—■—) receptor contents; the arrow indicates the $P = 0.05$ limit for the statistical significance; the number of amplified HER-2/*neu* tumours according to the cut-off value is indicated in brackets.

this study was to try to understand the discrepancies in the associations reported on in the literature, the uncorrected $P$ values could be used considering that each test was performed by a separate investigator and was therefore unique. This simulation has highlighted how the lack of standardisation of molecular biology techniques might contribute to some of the discrepancies reported in the relevant literature (Allred *et al.*, 1991). The meaning of these differences may solely be related to technical drawbacks or insufficiencies in the statistical analysis but may also indicate differences in the prognostic value according to the strength of the hybridisation signal, i.e. the number of copies of the gene, or may just be informative on the severity of the DNA disorders.

These were obviously not the only pitfalls of these analyses. Neither the slot nor the Southern blotting is able to take into account the heterogeneity of the tumour samples. In addition, as previously shown, the population selection might reveal new correlations. However, it clearly emphasises the need for a technical agreement based on inter centre studies to define precisely what actually is amplification. Another possibility is to standardise the statistical methodology to apply and the way to report the results.

Yours etc,

J.J. Pavy[1]
F. Descotes[2]
& G.L. Adessi[2,3]*
[1]Service de Radiothérapie et Oncologie,
[2]Service de biochimie Biologie Moleculaire,
CHU Jean Minjoz,
25030 Besancon Cedex, France,
[3]Unité de Biochimie Hormonale et des Régulations,
INSERM U198, 240 route de Dole,
25000 Besancon, France.
*To whom correspondence should be addresed

**References**

ALLRED, D.C., TANDON, A.K., CLARK, G.M. & MCGUIRE, W.L. (1991). HER-2/*neu* oncogene amplification and expression in human mammary carcinoma. *Biochem. & Molecular Aspects of Selected Cancers*, **1**, 75–97.

ALTMAN, D.G. (1992). Categorising continuous variables. *Br. J. Cancer*, **64**, 975.

DESCOTES, F., PAVY, J.J. & ADESSI, G.L. (1993). Human breast cancer: correlation study between HER-2/*neu* amplification and prognosis factors in a non selected population. *AntiCancer Res.*, (in press).

HILSENBECK, S.G., CLARK, G.M. & MCGUIRE, W.L. (1992). Why do so many prognostic factors fail to pan out? *Breast Cancer Res. Treat.*, **22**, 197–206.